# Aspects épidémiologiques des accidents vasculaires cérébraux (AVC) aux urgences de l'institut de cardiologie d'Abidjan (ICA)

**DOI:** 10.11604/pamj.2015.21.160.6852

**Published:** 2015-06-25

**Authors:** Yves N'da Kouakou N'goran, Fatou Traore, Micesse Tano, Kouadio Euloge Kramoh, Jean-Baptiste Anzouan Kakou, Christophe Konin, Maurice Guikahue Kakou

**Affiliations:** 1 Service des Urgences, Institut de Cardiologie d'Abidjan, Abidjan, Côte d'Ivoire

**Keywords:** Epidémiologie, AVC, urgences, cardiologie, Epidemiology, CVA, emergencies, cardiology

## Abstract

**Introduction:**

L'objectif de notre étude était de décrire les caractéristiques sociodémographiques et les Facteurs de Risque cardio-Vasculaires (FRV) des patients admis pour accidents vasculaires cérébraux (AVC) dans un service autre que celui de la neurologie.

**Méthodes:**

Étude transversale rétrospective sur une période de 2 ans (janv. 2010 et déc. 2011), réalisée aux urgences de l'institut de cardiologie d'Abidjan.

**Résultats:**

Il s'agissait de 176 adultes avec un âge moyen de 60 ans, une prédominance féminine. Les facteurs de risque majeurs retrouvés étaient l'hypertension artérielle dans 86,4% des cas, le diabète dans 11,4% des cas, le tabagisme dans 2,2% des cas. Les motifs de consultation étaient la perte de connaissance dans 36,4% des cas, l'hémiplégie dans 31,8% des cas, les céphalées dans 17,4% des cas, les vertiges dans 10,9% et les palpitations dans 2,2% des cas. La tension artérielle systolique moyenne était à 174 mmHg, la tension artérielle diastolique moyenne était à 105 mmHg et la pression pulsée moyenne était à 70 mmHg. Les AVC étaient associés à une arythmie complète par fibrillation auriculaire dans 11,4% des cas. Les AVC ischémiques représentaient 84,1%. L’évolution aux urgences a été marquée par un décès dans 17% (30) des cas.

**Conclusion:**

Les AVC constituent un problème majeur de santé publique. Malgré sa prédominance féminine, ils (AVC) touchaient 44% des hommes dans notre étude lorsqu'on sait qu'en Afrique l'activité sociale repose sur les hommes. Ils restent une pathologie grave par la forte létalité.

## Introduction

Les accidents vasculaires cérébraux (AVC) sont la deuxième cause de mortalité dans le monde et dans les pays en voie de développement (PVD), derrière les maladies cardio-vasculaires, devant les maladies infectieuses, notamment les infections pulmonaires ou diarrhéiques, la tuberculose, le sida ou le paludisme [1]. Ils sont parfois liés à une mauvaise hygiène de vie (tabagisme, obésité…), mais d'autres circonstances étiologiques existent notamment l′hérédité et certaines maladies spécifiques (hypertension artérielle, hypercholestérolémie, fibrillation auriculaire, troubles de la coagulation sanguine) [2]. En Côte d'Ivoire il est estimé que 9,3% des décès en milieu hospitalier public chez les sujets de 45 à 69 ans sont dus aux AVC qui sont un problème de santé publique [3]. L'objectif de notre étude était de décrire les caractéristiques sociodémographiques et les Facteurs de Risque cardio-Vasculaires (FRV) des patients admis pour AVC dans un service autre que celle de la neurologie afin de contribuer à une meilleure identification des cibles de la prévention des AVC.

## Méthodes

Il s'agissait d'une étude non exhaustive, portant sur 176 patients admis dans le service des urgences de l'institut de Cardiologie d'Abidjan (ICA); du 1^er^ Janvier 2010 au 31 Décembre 2011 et répondant au critère inclusif: malade de tout âge et des deux sexes admis pendant la période d’étude pour accident neuro vasculaire documenté par un scanner cérébral systématique. Ont été exclus de l’étude les AVC non documentés par une tomodensitométrie (TDM) cérébrale. Le diagnostic de l'accident vasculaire cérébral était clinique et para clinique: Clinique: tout patient présentant une anomalie clinique neurologique durant plus de 24 heures; para clinique: reposant sur des critères scannographiques; l'AVC hémorragique cérébral était retenu en présence d'une hyperdensité parenchymateuse; l'AVC ischémique était retenu lorsque le scanner était normal précocement ou lorsqu'il révélait une hypodensité désignant un territoire vasculaire artériel. En plus de la tomodensitométrie systématique d'autres examens seront réalisés en urgence pour l'approche étiologique: électrocardiogramme de repos, glycémie, créatininémie et hémogramme. Les tests statistiques utilisés étaient le test Epi Info.4 et Epi Data 3.1.

## Résultats

**Clinique**: sur 6975 malades admis aux urgences de l'ICA, 279 étaient admis pour AVC soit une prévalence de 4% dont 176 (63%) répondaient (Tableau 1) aux critères d'inclusion. L’échantillon se répartissait en 99 femmes (56%) et 77 hommes (44%) (Sex ratio de 1,2) et l’âge moyen était de 60 ans (32-86 ans). Les facteurs de risque majeurs étaient dominés par l'hypertension artérielle suivie du diabète et du tabac respectivement 86,4%; 11,4% et 2,2%. Les motifs de consultation étaient essentiellement la perte de connaissance dans 36,4% des cas, l'hémiplégie dans 31,8% des cas, les céphalées dans 17,4% des cas, les vertiges dans 10,9% et les palpitations dans 2,2% des cas. A l'examen clinique, ils avaient tous un déficit moteur, la tension artérielle systolique moyenne était à 174 mmHg (120-250), la tension artérielle diastolique moyenne était à 105 mmHg (76-140) et la pression pulsée moyenne était à 70 mmHg (40-110).

**Tableau 1:** Caractéristiques des patients

Variables	Fréquence	%
**AGE**		
Moyenne: 60 ans		
Minimum: 32 ans		
Maximum: 86 ans		
30 -39 ans	8	4.5
40 -49 ans	38	21.5
50 -59 ans	49	28
60 -69 ans	42	23.8
≥ 70 ans	39	22.2
**Sexe**		
Masculin	77	44
Féminin	99	56
**Antécédents**		
HTA	152	86.4
Diabète	20	11.4
Tabagisme	4	2.2
**Type AVC**		
Ischémique	148	84.1
Hémorragique	28	15.9
**Evolution immédiate**		
Décès	30	17

**Para clinique**: l’électrocardiogramme objectivait une arythmie complète par fibrillation auriculaire (AC/FA) dans 11,4% des cas, une hypertrophie auriculaire gauche dans 40% des cas et une hypertrophie ventriculaire gauche dans 89% des cas. L'AVC ischémique était le type lésionnel dominant constituant plus de 3/4 des lésions (84.1%%) dont 65% de lésion ischémique au scanner cérébral et 19.1% de scanner normal et les AVC hémorragiques 15,9% des cas.

**Evolution immédiate**: les patients qui avaient un AVC hémorragique, étaient tous transférés dans un service de réanimation et les AVC ischémiques étaient hospitalisés soit à l'institut de cardiologie (14%) ou dans un service de neurologie (67,4%). L’évolution aux urgences a été marquée par un décès dans 17% (30) des cas.

## Discussion

Cette étude rétrospective réalisée au service des urgences de l'ICA, nous a permis de constater un âge moyen des patients admis pour AVC à 60 ans; déjà décrite en Côte d'Ivoire [3]. Cet âge moyen est proche de celui d'autres africains [1, 4–7] variant de 44,5 ans à 61 ans ou d'une population Américaine à prédominance noire [8] avec 62 ans. Des auteurs, qui ont comparé la moyenne d’âge des AVC, entre les sujets de race différente, estiment que chez les sujets de race blanche cet âge moyen est de 77,5 ans et que 80,7% des sujets après 70 ans ont un AVC. Chez les latino-américains, ces deux chiffres étaient respectivement estimés à 65,4 ans et 38,5% alors que chez les sujets de race noire, ils étaient estimés à 69,5 ans et 49,8% [3]. La prédominance était féminine dans notre étude avec un sex ratio de 1,2. En Côte d'Ivoire une prédominance masculine a été déjà décrite [3]. En effet même dans la littérature africaine, il existe une variabilité de la prévalence selon le sexe, elle est soit féminine soit masculine: la majorité des études était en faveur d'une prépondérance masculine avec un ratio compris entre 1,3 et 1,5 [5, 6, 9], des ratios à 2 ont été décrits [1], de même qu'une prépondérance féminine avec des ratios compris entre 0,82 et 0,97 [10, 11]. Les facteurs de risque majeurs étaient dominés par l'hypertension artérielle suivie du diabète et du tabac respectivement 86,4%; 11,4% et 2,2%. Les dyslipidémies n'ont pas été mentionnées parce que leur dosage ne fait pas partir des examens réalisés en urgence à l'ICA. L'hypertension comme principal facteur de risque est décrite partout, que ce soit en Afrique [1, 4, 12–14] comme dans les pays développés [15]. La fréquence des facteurs de risque serait différente chez les sujets de race noire avec successivement HTA, tabagisme, diabète, cardiopathie alors que chez les sujets de race blanche ce serait plutôt tabagisme, HTA, cardiopathie, alcoolisme, diabète [15].

Les motifs de consultation retrouvés (perte de connaissance, l'hémiplégie, les céphalées ‘) dans notre étude ont été déjà écrites dans la littérature [12, 16]. L’électrocardiogramme objectivait une arythmie complète par fibrillation auriculaire (AC/FA) dans 11,4% des cas, une hypertrophie auriculaire gauche dans 40% des cas et une hypertrophie ventriculaire gauche dans 89% des cas. Ces principaux troubles retrouvés étaient les mêmes observés par Damourou Et Coll [12] qui a trouvé 19.78% d'AC/FA au Togo, Toure [17] 15.1% d'AC/FA au Sénégal et SRAÏRI [18] au Maroc en 2000 avait signalé 13,3% de cas dans sa série. Dans notre étude, nous avons observé une prépondérance des AVC ischémiques (AVC I) par rapport aux AVC hémorragiques (AVCH). En effet de nombreuses études ont montrées que les AVCI étaient beaucoup plus fréquents que les AVCH. C'est le cas en Mauritanie [19], au Nigeria [20] et dans les pays développés comme l'Espagne [21] et la Grèce [22]. Cependant, la prédominance des AVCI par rapport aux AVCH tend à se réduire de plus en plus voire à s'inverser depuis l'avènement de l'imagerie par scannographie en Afrique. Beaucoup d'hématomes intracérébraux minimes donnent des tableaux cliniques et évolutifs identiques à ceux des AVCI et Seule l'imagerie peut permettre de trancher d'où la limite de la clinique [17]. Notre létalité (17%) est concordante avec celle observée (10 à 60%) en hospitalisation africaine [4, 12, 16, 17], plus élevée qu'en Occident où l'admission rapide en neurologie ou mieux en unité neurovasculaire a considérablement réduit la mortalité des AVC [23, 24].

## Conclusion

Les accidents vasculaires cérébraux constituent un problème majeur de santé publique. Malgré sa prédominance féminine, ils (AVC) touchaient 44% des hommes dans notre étude lorsqu'on sait qu'en Afrique l'activité sociale repose sur les hommes. Les facteurs de risque cardiovasculaires sont dominés par l'HTA, le diabète et le tabac. Les AVC surtout ischémiques étaient souvent associés à une AC/FA dans notre étude. La prise en charge de cette association étant difficile, la prévention reste la seule mesure efficace. Cette prévention doit être primaire et doit avoir pour cible l'hypertension artérielle et les facteurs de risque cardiovasculaires. Ils restent une pathologie grave par la forte létalité dans notre étude.
